# Perceptions of homeopathy in supportive cancer care among oncologists and general practitioners in France

**DOI:** 10.1007/s00520-021-06137-5

**Published:** 2021-03-24

**Authors:** J. L. Bagot, I. Theunissen, A. Serral

**Affiliations:** 1 Main General Practice Surgery, Strasbourg, France; 2 Department of Integrative Medicine, Saint Vincent Hospital Group, Toussaint Hospital, Strasbourg, France; 3 Institut Rafaël-Maison de l’Après-Cancer, Levallois-Perret, France; 4 Breast Cancer Clinic, CHIREC Delta Hospital, Brussels, Belgium; 5 Laboratoires Boiron, Messimy, France

**Keywords:** Cancer patient, General practitioner, Homeopathy, Integrative oncology, Oncologist, Supportive care in oncology

## Abstract

**Objectives:**

In France, homeopathy is the most frequently used complementary therapy in supportive care in oncology (SCO); its use is steadily increasing. However, data is limited about the perception and relevance of homeopathy by oncologists and general practitioners (GPs) both with and without homeopathic training (HGPs and NHGPs, respectively). Our aim was to evaluate French physicians’ perceptions of homeopathy to clarify its place in SCO through two original observation survey-based studies.

**Materials and methods:**

Two cross-sectional surveys of French physicians were conducted involving (1) 150 specialist oncologists; (2) 97 HGPs and 100 NHGPs. Questions evaluated physician attitudes to homeopathy and patterns of use of homeopathic therapies in patients requiring SCO. Survey responses were described and analyzed on the basis of physician status.

**Results:**

Ten percent of oncologists stated they prescribe homeopathy; 36% recommend it; 54% think that homeopathy is potentially helpful in SCO. Two-thirds of the NHGPs sometimes prescribe homeopathy in the context of SCO and 58% regularly refer their patients to homeopathic doctors. HGPs have a positive perception of homeopathy in SCO.

**Conclusions:**

Homeopathy is viewed favorably as an integrated SCO therapy by the majority of French physicians involved with cancer patients—oncologists and GPs. Symptoms of particular relevance include fatigue, anxiety, peripheral neuropathy, sleep disturbance, and hot flashes. In such clinical situations, response to conventional therapies may be suboptimal and homeopathy is considered a reliable therapeutic option. These two studies highlight the fact that homeopathy has gained legitimacy as the first complementary therapy in SCO in France.

**Supplementary Information:**

The online version contains supplementary material available at 10.1007/s00520-021-06137-5.

## Introduction

Supportive care in oncology (SCO) includes all the necessary care and support given to patients with cancer alongside specific cancer therapy throughout the course of their disease [1–3]. Alleviation of physical and psychological symptoms and of treatment side effects through a global approach is key, placing individual patient needs, well-being, and quality of life at the core of clinical care.

In addition to conventional therapies, complementary and alternative medicine (CAM) is increasingly used by cancer patients [4–6]. In European countries, homeopathy represents one of the most frequently used CAM therapies in cancer care [7–9], and in France homeopathy is considered an established medical product [10]. Data suggests that homeopathy is the most common supportive care treatment for cancer patients [11–17]. In the VICAN 5 study, which examined a wide range of data on cancer survivors 5 years after diagnosis, 21.4% of survey participants reported use of non-conventional medicines, 59.6% of whom used homeopathy (including 15.9% using homeopathy exclusively for cancer-related reasons) [17].

While studies have evaluated attitudes toward CAM use in SCO by healthcare professionals and specialist oncologists [18–22], data specifically examining awareness, perceptions, and acceptance of homeopathy by physicians caring for cancer patients is limited. We wished to evaluate expectations, experiences, and opinions of doctors about homeopathic medicine in SCO in France through cross-sectional surveys of key physician groups.

Two separate cross-sectional, observational, descriptive studies were conducted using online questionnaires to collect data. We addressed two groups of physicians involved in cancer care: (1) specialist oncologists (medical oncologists, hematologists and radiation oncologists), and (2) two groups of general practitioners (GPs)—“homeopathic GPs” (HGPs) trained in homeopathy and “non-homeopathic GPs” (NHGPs) who had no specific knowledge or training in homeopathy. The aim was to evaluate:

1. Oncologists’ levels of satisfaction with conventional supportive care treatments as well as their knowledge and attitude toward the role of homeopathic therapy and its most appropriate indications in SCO.
2. The use of homeopathic medicines in cancer patients by HGPs and NHGPs in primary care and to explore the differences in their attitudes toward use of these medicines in SCO.

Note that while the broader theme of these studies was that of exploring attitudes and use of homeopathy in specialist or primary care settings, these surveys/studies were entirely independent of each other, and indeed were conducted at different times (2014 and 2017 respectively), and for logistical reasons, each conducted with the assistance of a different market research agency. In addition, it is not our aim to compare and contrast oncologists and GP responses. The purpose of this manuscript is to report on what we believe are relevant findings from each of these studies that may inform the wider medical community.

## Materials and methods

### Specialist oncologist study

A cross-sectional descriptive survey was developed based upon a set of key questions discussed by the authors at a preliminary meeting; a final survey format was developed and subsequent administration and data collection performed by a market research agency (AplusA, 92641 Boulogne Billancourt Cedex, France). The final survey (administered as an online questionnaire) included a suite of binary (yes/no) or scalar multiple-choice questions and “freestyle answer” questions. Data was collected across a range of domains evaluating participant satisfaction with conventional supportive care treatments and their knowledge of and attitudes toward homeopathic therapy in SCO. Selected questions from the oncologists’ survey directly relevant to the findings we report are shown in Supplementary Table 1.

**Fig. 1:**
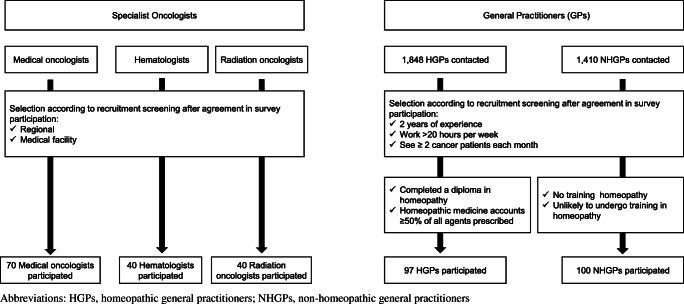
Flow chart of the designs of the two studies

Potential participants were identified from an established database of 2300 specialist oncologists (out of a total of 2369 currently practicing within France). Provisional screening was performed using a random sampling approach, stratified on the basis of age, gender, oncology specialty (medical oncology, hematology and radiation oncology), medical institution (state-funded or other, teaching hospital, etc.) and region. Potential participants were invited and if agreeable recruited. The aim was to recruit a representative sample of 150 specialists from across these three principal oncology specialties, and recruitment ceased after 150 participants had agreed (Fig. 1). Participating physicians received a modest financial honorarium for completing the questionnaire.

### GP study

For this study, survey development/administration was similar to the process used for specialist oncologists, although a different agency (Axess Research, 69760 Limonest, France) was used for survey administration and data collection. The final survey, presented as an online questionnaire, included a suite of multiple-choice or “freestyle answer” questions which addressed their attitudes toward and clinical use of homeopathy agents as supportive care for their patients with cancer. Selected questions from the GP survey directly relevant to the findings we report are shown in Supplementary Table 1.

Recruitment followed a similar process to that reported in previous studies [13]. Participating GPs were recruited from two databases, one for NHGPs and one for HGPs. From these, a total of 1848 HGPs and 1410 NHGPs were contacted and screened for suitability. Recruitment targets were to include a total of 100 GPs in each group. Eligible participants were identified on the basis of specific criteria. All were required to have a minimum of 2 years’ experience, work more than 50% of a conventional working week, and have consultations with at least two cancer patients each month. NHGPs were required to have undertaken no training in homeopathy and to indicate that they were unlikely to undergo such training; HGPs were required to have completed training in homeopathic medicine and have a predominantly homeopathic therapy clinical prescribing pattern, i.e., homeopathic medicine accounts for ≥50% of all medicines prescribed (Fig. 1). Participating physicians received a modest financial honorarium for completing the questionnaire.

### Data analysis

Our analyses were primarily descriptive. For both surveys, responses were tabulated according to physician category or pooled across categories as appropriate. Continuous variables were expressed as their mean ± standard deviation (SD), or minimum and maximum; discrete variables were expressed as group size and percentage. Quantitative variables were analyzed using the Z-test of the gap reduction for mean comparisons. Although no formal power calculations were performed, some statistical analyses were undertaken; categorical or discrete variables were analyzed using the chi-square test or Student’s *t*-test. All statistical tests were two-sided with *p* values < 0.05 considered statistically significant. All data were anonymized and stored and analyzed using Microsoft Excel or Sphinx software.

## Results

### Oncologists’ perspective on homeopathic therapy in supportive cancer care

**Fig. 2:**
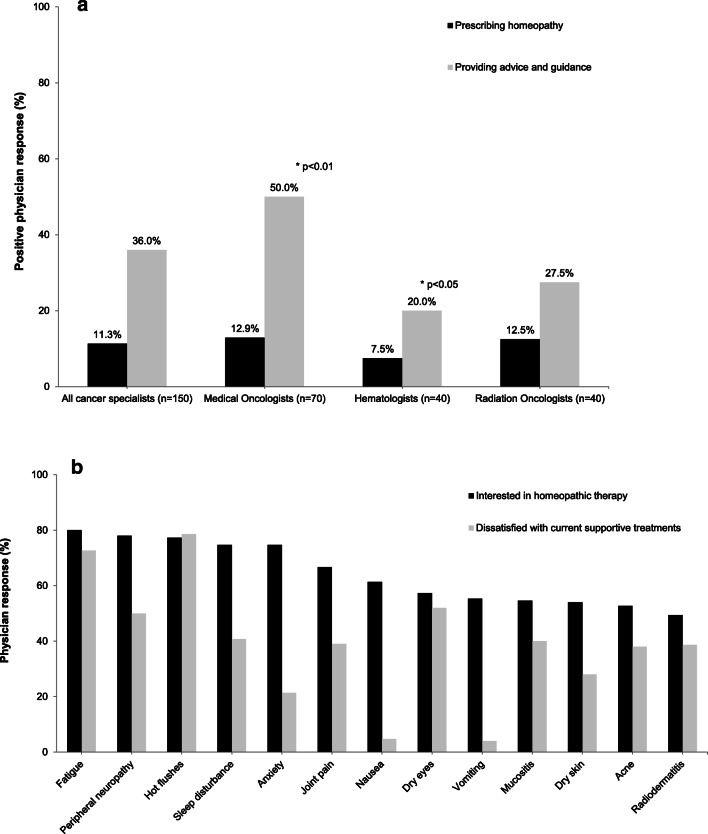
Specialist oncologists’ perspectives on homeopathic therapy as supportive care treatment. **a** Proportion of specialist oncologists either prescribing or providing patient guidance on homeopathic therapy in their current practice. **b** Relative dissatisfaction with current available treatments and interest in homeopathic therapy in the management of specific symptoms/side effects of cancer therapy

Between June 24th and August 1st 2014, 150 specialist oncologists completed the survey: 70 medical oncologists, 40 hematologists and 40 radiation oncologists. Respondents were from a range of clinical settings; although between-group differences were not formally evaluated, all three specialty groups were broadly comparable in terms of age, gender and institutional setting. The characteristics of these physicians are presented in Table 1. When asked about their current practice, 11% of respondents reported actively prescribing homeopathic therapy as supportive care, while 36% would recommend it as a treatment and provide guidance and patient referral to homeopathic physicians. Some differences in practice were seen across different oncology specialties; while the proportion who actively prescribed homeopathic therapy was broadly similar in each specialty (ranging between 7.5 and 12.9%), medical oncologists were more likely to refer patients for homeopathic treatment than other specialties (50%), while hematologists were less likely to do so (20%; *p* < 0.01 and *p* < 0.05 respectively) (Fig. 2a). The majority of oncologists surveyed indicated an interest in the use of homeopathic treatment for a range of side effects associated with cancer treatment: fatigue (80.0%), peripheral neuropathies (78.0%), hot flashes (77.3%), sleep disorders (74.7 %), and anxiety (74.6%). For most of these (and others), dissatisfaction with existing management options was high (Fig. 2b).

**Table 1:** Characteristics of participating physicians specialized in oncology

Characteristic	All oncology physicians (*N* = 150)	Medical oncologists (*N* = 70)	Hematologists (*N* = 40)	Radiation oncologists (*N* = 40)
Age (mean ± SD)	44.9 ± 8.0	44.1 ± 7.7	45.8 ± 8.3	45.4 ± 7.9
Gender (*n* (%))				
Men Women	99 (66.0) 51 (34.0)	44 (62.9) 26 (37.1)	27 (67.5) 13 (32.5)	28 (70.0) 12 (30.0)
Medical facility type (*n* (%))				
State-funded center (teaching) State-funded center (non-teaching) Oncology center Private hospital	66 (44.0) 45 (30.0) 18 (12.0) 21 (14.0)	28 (40.0) 22 (30.4) 10 (14.3) 10 (14.3)	28 (70.0) 12 (30.0) 0 0	10 (25.0) 11 (27.5) 8 (20.0) 11 (27.5)
Pathology of specific interest (%)				
Breast cancer Lung cancer Colorectal cancer Prostate cancer Others cancers		29.1 17.2 20.5 15.4 17.8		

*N*, total number of physicians completing the survey; *n,* number of physicians with each characteristic; *SD*, standard deviation

More than half (54%) indicated their belief that homeopathic medicines are a satisfactory option in the treatment of certain side effects of cancer therapy. Simplicity of use was considered a positive attribute (64.6%), as was the involvement of patients in their treatment (67.3%). Most (86%) expressed an interest in further information on clinical studies of homeopathic therapies in cancer patients and 88% an interest in potential drug interactions with conventional treatments; this was of particular interest to radiation oncologists (95%). Protocols for use were considered helpful to inform decision-making by 70% of respondents.

### General practitioners’ experience with homeopathic therapy in supportive cancer care

The GP survey was conducted between June 6th and July 4th 2017, with responses collected from 197 physicians: 97 HGPs and 100 NHGPs. Most were engaged in full-time clinical practice, and highly experienced (with most reporting over 20 years of clinical experience). A greater proportion of NHGPs provided services reimbursed under regulated national tariffs than HGPs (88% vs. 52%; *p* < 0.01). Characteristics of GP respondents are presented in Table 2.

**Table 2:** Characteristics of participating general practitioners

Characteristic		HGPs (*N*=97)	NHGPs (*N*=100)	*p*-value
Clinical experience, years, mean ±SD		28 ± 7.3	21 ± 11.9	0.01^a^
Work time (*n* (%))	50 to 99% of the time	28 (29)	12 (12)	0.003^b^
	100%—full time	69 (71)	88 (88)	
Reimbursement sector of activity (*n* (%))^c^	Sector 1 (regulated “fixed fee”)	50 (52)	88 (88)	<0.01^b^
	Sector 2 (unregulated “free-billing”)	45 (45)	12 (12)	
	Other	2 (2)	0 (0)	
% of prescriptions with homeopathic medicines (mean ± SD)		88 ± 12.6	18 ± 23.2	<0.0^a^
Number of consultations where supportive cancer care is addressed per month (mean ± SD)		19 ± 37.6.	16 ± 16.7	0.54^a^

^a^*p*-value determined by Student’s *t*-test

^b^*p*-value determined by Chi-square test

^c^GPs in sector 1 provide services and are reimbursed on the basis of regulated/statutory national tariffs; in sector 2, GPs can set their own fees for treatment and can claim for additional items of service

*N*, total number of practitioners completing the questionnaire; *n*, number of practitioners with each characteristic; *SD*, standard deviation

**Fig. 3:**
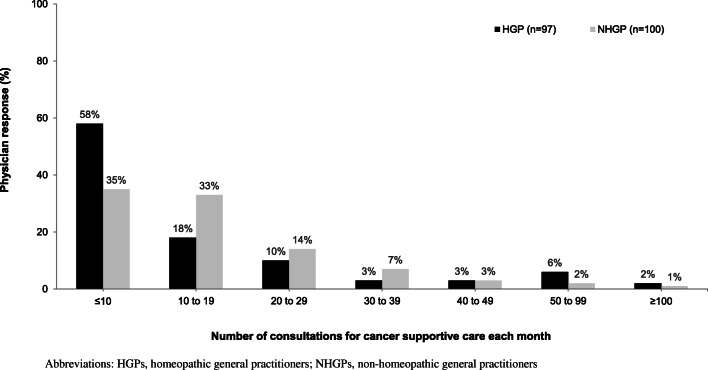
Supportive care in oncology as a component of GPs’ clinical time. No significant differences were found

Supportive care for cancer patients represented an important aspect of clinical practice in both groups (an average of 17 consultations per month), with some GP practices partially or exclusively dedicated to such care (Fig. 3). No significant differences in number of consultations for supportive cancer care were seen between the HGP and NHGP groups. A higher proportion of patients whose reason for consultation was the management of side effects associated with cancer treatment was reported by HGPs than NHGPs (53% vs. 29%; *p* < 0.05).

More HGPs than NHGPs reported being familiar with SCO (95% vs. 76%, respectively; *p* < 0.05).

Perspectives on what constitutes SCO differed between groups: when asked to define SCO, 58% of HGPs considered it as the management of cancer treatment side effects, compared with 18% of NHGPs (*p* < 0.05). Psychological support was considered an important aspect of SCO by 28% of HGPs and by13% of NHGPs (*p* < 0.05). However, 30% of NHGPs indicated that an important component of SCO is to provide comfort, well-being and improvement in the quality of life, which clearly could include an element of psychological support.

NHGPs’ perception of homeopathy in SCO was positive (72%), including 19% very positive, with 64% prescribing homeopathic therapies within the framework of SCO; the most frequent indications were the management of fatigue, anxiety and nausea. The principal reason for NHGPs not prescribing homeopathy in SCO was a lack of education in its use and benefits (indicated by 47% of NHGPs). Referral of patients by NHGPs to an HGP for SCO consultation was frequent (reported by 58%), while 83% were in favor of further development of the use of homeopathic therapies in SCO.

**Fig. 4:**
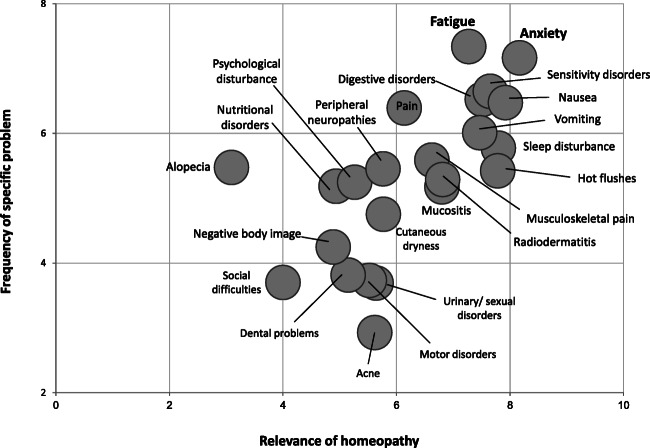
Utility of homeopathic therapy in clinical practice of homeopathic general practitioners (HGPs). Matrix showing the frequency of specific symptoms and relevance of homeopathic therapy in the experience of HGPs

HGP respondents indicated a positive attitude toward homeopathy; the principal reasons for its use in cancer patients are its safety and effectiveness. For addressing specific symptoms, homeopathy was considered by HGPs to be most reliable in the treatment of fatigue and anxiety; nausea, vomiting, sleep disturbance, hot flashes, and sensitivity disorders were also considered important (Fig. 4). In contrast, other aspects (alopecia, social difficulties and negative body image) were not considered particularly relevant to homeopathic therapy. Most HGPs (87%) reported prescribing homeopathy in conjunction with conventional medicines. Prescription details were not uniformly communicated to the specialist oncologist responsible for treating the patient’s cancer, with only 26% of HGPs reporting doing this “often” and 34% doing so “sometimes.” More than half of HGPs (54%) indicated their willingness to be responsible for a homeopathic consultation in an oncology hospital or clinic if requested to do so. In order to develop and improve their SCO prescriptions, the majority reported the value that additional clinical studies on homeopathic therapy in supportive cancer care would bring, along with avenues for education such as workshops.

## Discussion

In these cross-sectional observational descriptive studies, we surveyed specialist oncologists and GPs (conventional or trained in homeopathy) to establish current attitudes to and use of homeopathic therapies in SCO in France. Although a number of studies indicate that homeopathy is the complementary medicine most commonly used by cancer patients in France [11–17], data is more limited for factors which may influence clinical decision-making for recommending or prescribing homeopathic therapies as SCO by treating physicians. It was this, rather than a formal evaluation and analysis of specific prescribing patterns, which was the main focus and purpose of the studies we report.

Our results show that homeopathy in SCO was viewed positively by the majority of physicians surveyed. More than half of oncologists considered homeopathy to be a satisfactory option in the management of treatment side effects, with a high level of interest in its use in specific circumstances such as fatigue, peripheral neuropathy, hot flashes, sleep disorders and anxiety, where in many cases they reported dissatisfaction with current conventional treatments. Simplicity of use also rated highly. There was a clear need for further information from clinical studies formally evaluating the effectiveness of homeopathy in SCO to increase confidence and awareness. These findings are in general agreement with other surveys evaluating attitudes of oncologists and other healthcare professionals (HCPs) working in tertiary care toward use of CAM in SCO in other European countries (including Germany and Switzerland) [18–22]. An overall theme across most studies is that greater education and training is critical to optimizing CAM use in cancer care.

There is increasing awareness of the concept of integrative oncology care, where a range of CAM therapies are used alongside conventional cancer treatments in a patient-centered approach [23]. Adoption of integrated care into routine clinical cancer care services in oncology centers is increasing in Europe [9], and it seems likely that such integrative care will become more widely available. Homeopathy is an important component of integrative care in most European centers [9] and, given its frequent use by cancer patients in France, would seem to be one of the most important integrative therapies. Advocacy and educational support for homeopathy in integrated care is available from the International Homeopathic Society for Supportive Care in Oncology (IHSSCO), established in 2016, whose aim is to facilitate and develop the practice, teaching, research, and promotion of homeopathic therapy as part of SCO [24]. IHSSCO has identified 15 hospitals offering homeopathic consultation in SCO across France and has published therapeutic recommendations developed by expert consensus to guide use [25]. IHSSCO congresses and educational activities are well attended by HGPs (and other HCPs with training or interest in homeopathy).

From the GP perspective, SCO consultations represent an important component of clinical practice, reflecting the value of GPs in the multidisciplinary care of cancer patients. Among NHGPs, interest in homeopathy was also high (higher than that of specialist oncologists). In addition, two-thirds of NHGPs prescribe homeopathic therapies in an SCO context, despite their having had no formal training, while more than half refer their patients to an HGP for an SCO consultation. This perhaps reflects their recognition of the need for homeopathic expertise to include homeopathy in a truly multidisciplinary approach to supportive cancer patient care. HGPs, as expected given their training and clinical experience in homeopathy, had the most positive approach to homeopathic therapy in SCO, chiefly due to their belief in its safety and effectiveness. Over half of the HGPs indicated their interest in participating in dedicated homeopathy clinics in SCO; as stated above, such clinics are increasing in France. Like the other physician groups examined, HGPs also highlighted a desire for further information on clinical studies and continuing medical education activities on homeopathy in SCO.

We found that there was a relatively low level of communication between HGPs and the treating specialist oncologist regarding prescription of homeopathic therapies as SCO; this was surprising and we can only speculate about the reasons for this (e.g., fear of adverse comments or lack of time). The demand from specialists for additional information on homeopathy would suggest that they would welcome such information, and HGPs should consider this to be an important aspect of multidisciplinary SCO. We hope that such communication will increase in the future.

HGPs ranked treatment of fatigue, anxiety, sleep disturbance, and hot flashes highly as specific components of SCO that benefit from homeopathic medicines (and which in their experience are often unmet with conventional treatment options); these correspond to our own clinical experience. Fatigue is perceived as a major indication for homeopathy in SCO. In their pragmatic randomized controlled trial, Frass et al. demonstrated that adding homeopathic therapy to conventional supportive treatments was associated with significant improvement in fatigue scores compared with conventional treatment alone (*p* < 0.001) [26]. Homeopathy should be considered a useful treatment option in such patients. Anxiety and sleep disorders contribute to a patient’s emotional status and general feeling of well-being. In the same trial, use of adjunctive homeopathy was associated with significant improvements in patient global health status and subjective well-being (*p* < 0.001). Significant improvements in social and emotional functioning (that could be considered proxy indicators of lower anxiety) and significant improvements in insomnia were also reported [26].

Iatrogenic hot flashes are an important side effect of some cancer treatments, notably hormonal treatment in breast cancer. Data indicates that homeopathy may be effective in reducing menopausal hot flashes, and studies in patients with breast cancer suggest that it may also be beneficial in reducing (i.e., improving) hot flashes scores with a positive impact on quality of life [27, 28]. Management of nausea and vomiting was also considered relevant; again, this matches our own experience, especially when persistent despite taking conventional antiemetics. Other symptoms were rated less highly by HGPs in terms of relevance to their use of homeopathy as SCO. Nevertheless, they are often reported as being of importance, and symptoms such as musculoskeletal pain have been shown to respond favorably to homeopathy [26]. In a recent observational study of use of adjunctive homeopathy in the prevention of musculoskeletal pain secondary to aromatase inhibitors, Karp et al. reported significant reductions in mean composite pain scores and lower analgesic consumption with homeopathy versus conventional treatment alone [29].

Our study has several limitations. The aim of both surveys was to evaluate perceptions and practices of physicians on integrating homeopathy into SCO. In this we surveyed specialist oncologists (in 2014) and GPs with and without a specific interest in homeopathy (in 2017). While these surveys reflect the views of these physician groups at that time, it is possible that physician perceptions, and use of homeopathy in SCO may have subsequently changed.

As with all such observational survey-based studies, there was a potential for selection bias among the physicians who completed the surveys. In both studies, we sought to reduce any such bias by recruiting a balanced representative sample from established physician databases. However, the potential for bias may remain, and our findings considered in this context. While the number of respondents across both studies was suitably large to extract relevant and interpretable data, no formal power calculations were performed. For GPs, we specifically recruited doctors with relevant current experience with SCO in clinical practice; although self-declared, we believe that the classification of GPs into the relevant HGP and NHGP groups was rigorous enough to allow some analysis and reporting of different attitudes and clinical practice regarding homeopathic SCO. Our observations are primarily descriptive and report physician attitudes and perceptions; we did not seek to examine prescribing patterns or attitudes toward specific agents, nor to analyze the use of relevant conventional treatments. Similar studies examining these specific aspects would provide important information on the “global” value of homeopathy within integrated SCO. Finally, our study investigated the role of homeopathy from the physician’s perspective. Clearly data on expectations and satisfaction from the patient’s perspective is also of considerable importance, especially as it has been reported that cancer patient’s motives for choosing and consulting with HGPs and NHGPs may differ [13].

On a broader note, while we recognize that other complementary integrative therapies are important aspects of integrative SCO, they were beyond the scope of the present manuscript. Similar studies looking at the real-world status and perceptions of other complementary therapies (such as acupuncture or phytotherapy) could be performed to examine their roles in contemporary SCO in France.

## Conclusion

A discussion about the value of homeopathy in SCO in France is taking place between healthcare professionals, leading to an active collaboration. The results from these two studies represent the first evaluation of the opinion of French doctors on the role and relevance of homeopathy in SCO.

The choice of integrative and complementary medicine is often cultural. In France, due to widespread acceptance by both doctors and patients [11–17], homeopathy is an important and legitimate component of the French integrative model. Key aspects are patient satisfaction, safety, low cost, ease of use, and absence of important drug interactions. This suggests that homeopathy is a valuable therapy in SCO, especially in patients for whom conventional therapeutic options fail. Further clinical studies examining the use of homeopathy in SCO will improve our knowledge of the specific role of homeopathic therapy in these circumstances.

## Supplementary Information

ESM 1 (DOCX 17.2 kb)

## Data Availability

N/A

## Code availability

N/A
