# Supplementary material for: Perceptions of homeopathy in supportive cancer care among oncologists and general practitioners in France
Source: Support Care Cancer. 2021 Mar 24;29(10):5873–81. doi: 10.1007/s00520-021-06137-5 (PMC8410724; doi:10.1007/s00520-021-06137-5)
Supplement: Supplementary file 1 — (DOCX 17.2 kb) [file 520_2021_6137_MOESM1_ESM.docx]

**Supplementary Table 1 Selected questions asked in the oncologist and GP surveys**

| **Oncologist survey** | |
| --- | --- |
|  | For homeopathy as a complementary therapy in supportive cancer care: Please specify if you 1) prescribe or 2) provide advice on or referrals to other practitioners or 3) do not prescribe nor provide advice or referrals. |
|  | Listed here are various side effects associated with cancer treatment. For each, indicate your satisfaction with the therapeutic solutions currently available: very satisfactory; somewhat satisfactory; somewhat unsatisfactory; or unsatisfactory:  Fatigue, peripheral neuropathy, hot flashes, sleep disturbance, anxiety, joint pain, nausea, dry eyes, vomiting, mucositis, dry skin, acne, and radiodermatitis. |
|  | For each of the following indications, please specify if you would be interested in a homeopathic therapeutic option: very interested; somewhat interested; not very interested; or not at all interested.  Fatigue, peripheral neuropathy, hot flashes, sleep disturbance, anxiety, joint pain, nausea, dry eyes, vomiting, mucositis, dry skin, acne, and radiodermatitis |
|  | Listed here are different opinions about homeopathy as a complementary therapy in supportive cancer care. For each, indicate your level of agreement: strongly agree, somewhat agree; somewhat disagree; or strongly disagree:  Use of homeopathic therapy is simple; Homeopathic medicines allow patients to get involved in their care |
|  | Listed here are various topics of information concerning homeopathic medicines. For each, please indicate your level of interest: very interested; quite interested; only a little interested; or not at all interested:  Different items proposed including: Clinical studies in cancer supportive care; potential interactions with conventional treatments; treatment protocols |
| **GP survey, some questions are asked to HGPs or NHGPs** | |
|  | Do you consider yourself to be familiar with supportive cancer care? |
|  | How do you define “supportive cancer care? [Note this was free-form question, with responses grouped into categories as follows]  Management of the side effects of principal cancer treatment  Medical care provided in parallel to cancer treatment  To provide patient comfort, well-being, and improve quality of life  To provide psychological support |
|  | What proportion of your patients with cancer attend for treatment of side effects related to their cancer treatment? |
|  | How do you perceive homeopathy in supportive cancer care? Very positive, quite positive, quite negative, very negative |
| for NHGP | Do you prescribe homeopathic medicines in supportive cancer care? Sometimes, often, never |
| for HGP | a) In the following list of side effects of cancer care, specify how often each of them is raised (by you or the patient) during the consultations with the cancer patients [list of principal side effects of cancer care; Fatigue, peripheral neuropathy, hot flashes, sleep disturbance, anxiety, joint pain, nausea, dry eyes, vomiting, mucositis, dry skin, acne, radiodermatitis, alopecia, and other general symptoms (including digestive disorders, nutritional disorders, psychological disturbance).], Very often, often, rarely, never  b) For the topics frequently address in consultation (very often, often), do you prescribe homeopathic medicines to answer this demand? Very often, often, rarely, never |
| for HNGP | If you do not prescribe homeopathic medicines in supportive cancer care please provide a reason? [free form answers subsequently categorised into groups; Lack of training / skills in their use in supportive cancer care; homeopathic medicines are not 100% reimbursed by State insurance; preference to refer to a homeopathic doctor; lack of knowledge on homeopathy; questionable effectiveness; other. |
| for NHGP | How often do you refer the cancer patients to other health professionals? Psychologist/ Acupuncturist/ Nurse/ Homeopathic general practitioner/ Pharmacist? Very often, often, sometimes, never |
| for NHGP | Are you in favour of greater development of the use of homeopathic therapy in supportive cancer care? Very favourable, favourable, unfavorable |
|  | Do you have direct communication with the oncologist responsible for the care of your patients with cancer? Often, sometimes, rarely, never |
|  | Do you provide the oncologist with details of your homeopathy treatment? Often, sometimes, rarely, never |
